# Association between Living with Patients with Dementia and Family Caregivers’ Depressive Symptoms—Living with Dementia Patients and Family Caregivers’ Depressive Symptoms

**DOI:** 10.3390/ijerph18084372

**Published:** 2021-04-20

**Authors:** Minah Park, Fatima Nari, Soo Hyun Kang, Sung-In Jang, Eun-Cheol Park

**Affiliations:** 1 Department of Public Health, Graduate School, Yonsei University, Seoul 03722, Korea; minahpark@yuhs.ac (M.P.); fatima@yuhs.ac (F.N.); kshyun@yuhs.ac (S.H.K.); 2 Institute of Health Services Research, Yonsei University, 50 Yonsei-ro, Seodaemun-gu, Seoul 03722, Korea; JANGSI@yuhs.ac; 3 Department of Preventive Medicine, College of Medicine, Yonsei University, 50 Yonsei-ro, Seodaemun-gu, Seoul 03722, Korea

**Keywords:** dementia, depression, family caregivers

## Abstract

Depression among family caregivers is becoming an increasingly important issue due to a growing elderly population. This study aimed to examine the association of living with a patient with dementia and family caregivers’ depressive symptoms, among Korean adults. This study used the data of 371,287 participants after excluding those who indicated having dementia themselves from the Korea Community Health Survey of 2018–2019. Depressive symptoms were measured using the Patient Health Questionnaire-9. Data were analyzed using multiple logistic regression. The rates of spouse caregivers having depressive symptoms were 9.4% and 10.8% among men and women, respectively. The odds ratio for risk of depressive symptoms among male and female spouse caregivers in comparison to non-caregivers was 2.65 and 2.28, respectively. In the subgroup analysis, the highest income group was associated with risk of depressive symptoms, with an odds ratio of 4.28 for men, and 3.02 for women. Having a patient with dementia in the family was significantly associated with family caregivers’ depressive symptoms. In particular, when the patient with dementia was a spouse, both women and men were likely to have depressive symptoms. To reduce the burden of caregivers, we need management policies and interventions for family caregivers.

## 1. Introduction

Dementia is a term that covers a wide range of conditions and variety of diseases [1], which include Alzheimer’s disease, vascular dementia and Parkinson’s disease [2]. Dementia is caused by abnormal brain changes, and affects daily life, independent functioning, behavior, feelings, and relationships. Dementia is present in approximately 10% of people aged 65 years and older [3]. Fifty million people live with dementia worldwide and the number is projected to increase to 152 million by 2050 [4]. The frequency of dementia in the Republic of Korea is between 7.2% and 8.2% among the urban Korean elderly population [5,6].

Depression is one of the most universal health issues [7]. Major depressive symptoms are not only a burden on the individual, but also places a considerable burden on the society [8]. In a 2015 study, depression was a leading cause of age-specific disability-adjusted life years (second in 20–24 years old, fourth in 25–39 years old, fifth in 40–44 years old, and sixth in 45–54 years old) [9]. In the Republic of Korea, mental health problems have become a national issue. The Korean Epidemiologic Catchment Area study, an epidemiological survey on mental health conducted every five years, has reported a gradual increase in the prevalence of depression (2001, 4.0%; 2006, 5.6%; 2011, 6.7%) [10].

As the elderly population grows, there will be a significant increase in dementia caregiving. Family caregivers end up filling the gaps that exist in the healthcare system and doing so can affect them physically, emotionally, socially, financially, and spiritually [11]. Dementia caregivers often experience elevated levels of stress, and are at increased risk for psychological disorders [12]. Over the last 25 years, the crisis of family caregivers of people with dementia has led to an increasing number of research studies designed to evaluate innovative strategies for helping families maintain a high quality of life for both caregivers and care-recipients alike [13]. The frequency of dementia in the Republic of Korea is between 7.2% and 8.2% among the urban Korean elderly population Many studies have examined many aspects the family caregivers’ health. For example, Jang et al. [14] analyzed the association between cohabitation status with a patient with dementia in the family and Son et al. [15] analyzed the predictors of burden and satisfaction in Korean adult child caregivers of older adults with dementia. However, these studies are limited by the generalization of family caregivers or examining only the adult child in caregiving. The purpose of this study, therefore, was to analyze the relationship of having a family member with dementia and family caregivers’ depressive symptoms using nationally representative data. A secondary purpose of this study was to examine this association stratified by sex.

## 2. Materials and Methods

### 2.1. Data

The data used in this study were obtained from the Korea Community Health Survey (KCHS), which was administered by the Korea Center for Disease Control and Prevention Agency (KDCA) in 2018 and 2019. The KCHS standardized questionnaires were developed by staff working at the KDCA and the Working Group of Health Indicators Standardization Subcommittee at 16 metropolitan and 253 region sites. The questionnaires cover many different topics including health behaviors, driving, quality of life (European Quality of Life-5 Dimensions, European Quality of Life-Visual Analog Scale, Korean Instrumental Activities of Daily Living), medical service utilization, chronic disease, and so on. The microdata (in the form of Statistical Analysis Software [SAS] files) and analytic guidelines can be downloaded from the KCHS website (9 October 2020).

### 2.2. Participants

The final data gathered were from 371,287 individuals (187,862 in 2018 and 183,425 in 2019). Data from both years were integrated and sampling weights were incorporated for the analysis. A total of 876 individuals who had dementia were excluded. Our study did not need to address any ethical concerns because the KCHS data is a secondary dataset that are available to the public domain and does not contain private information.

### 2.3. Variables

The variable of interest in this study was the family relation of the patient with dementia. The KCHS asked each individual “If one of your family members has dementia, who is it?” and the response options were multiple choice (Parent including In-Laws, Spouse, Sibling, Yourself, and Children).

The dependent variable that was used is the Patient Health Questionnaire-9 (PHQ-9). This is a self-report questionnaire that includes nine questions to evaluate depressive symptoms [16]. Used in primary care and other medical settings to screen depression, the cut-off score for major depressive symptoms is 10, with scores of 10 and above indicating presence of depressive symptoms [17]. With a total score of 27, each question is rated as 0 for “Not at all”, 1 for “For many days”, 2 for “more than a week”, and 3 for “almost daily”.

The independent variables included the survey year, sex, age, employment status, education level, marital status, family income, smoking habit, alcohol use, number of family members, and status of receiving basic living allowance. Age was divided into five categories: from 19 to 39, 40 to 49, 50 to 59, 60 to 69, and 70 or older. Survey year was divided into two categories: 2018 and 2019. Region was divided into two categories: metropolitan and rural. Education was divided into three categories: university or higher, high school, and middle school or less. Job status was divided into four categories: white (managerial, professional, or clerical), pink (services or sales) blue (manual labor) and unemployed. Marital status and receiving basic living allowance were divided into two categories: Yes and No. Current smoking status was divided into current smokers and none current smokers. Alcohol use was measured depending on whether the participant drank alcohol within a year and categorized as Yes and No. The variable for the number of family members living in the same household was divided into five categories: 1, 2, 3, 4, and 5+. Household income was divided into four categories: high (≥60,000,000 KRW), middle high income (≥35,000,000 KRW <60,000,000 KRW) middle-low income (>20,000,000 KRW <35,000,000 KRW), and low income (≤20,000,000 KRW).

### 2.4. Statistical Analysis

Chi-squared test was used to investigate the association between covariates and depressive symptoms. Multiple logistic regression analysis was used to assess the association between family caregiver status and depressive symptoms. The results were reported using odds ratios (ORs) and confidence intervals (CIs). The subgroup analysis was conducted to investigate the association according to stratified age, job, region, educational level, and income using a multiple logistic regression analysis. After we stratified the participants into subsets based on five variables, to examine how the relationship between family member with dementia and depressive symptoms differs from one characteristic to another. For instance, the probability of depressive symptoms could vary between those who have a white collar job to those who reported to be unemployed. The subgroup analysis could be useful in determining which characteristics of the participants serve as the effect modifier in explaining the relationship between family member with dementia and depressive symptoms. Differences were considered statistically significant at *p*-values of <0.05. The data were analyzed over the entire population and then stratified by sex using SAS 9.4 (SAS Institute Inc.; Cary, NC, USA).

## 3. Results

Table 1 presents the results of univariate analyses that examine the association between self-rated PHQ-9 score and the presence of patients with dementia in the family and each variable by sex. The overall depressive symptoms scores were higher in women than men (5.1% vs. 2.4%, respectively). The depressive symptoms rate was three times higher in both sexes when their spouse had dementia (10.8% vs. 9.4%). However, the PHQ-9 score was lower when the respondent’s parent had dementia (4.7% vs. 2.1%).

Table 2 reports the logistic regression results stratified by sex for the association between PHQ-9 and the relationship with the patient with dementia for all variables. For both men and women, those who had a spouse with dementia had increased odds of depressive symptoms (women: OR 2.28, CI 1.77–2.93, men: OR 2.65, CI: 2.06–3.32). However, only women showed increased odds of depressive symptoms when the parent was the patient with dementia (OR 1.47, CI 1.32–1.62).

Table 3 reports the subgroup analysis of the association between relation with dementia patient and caregiver’ depressive symptoms stratified by sociodemographic variables. For women who had a parent with dementia, all regions, income levels, and job status had a higher probability of depressive symptoms. Regardless of gender, when having a spouse with dementia, all regions, blue collar jobs or unemployed had a higher probability of depressive symptoms.

## 4. Discussion

This study used the KCHS to analyze the association between the presence of patients with dementia in the family and family caregivers’ depressive symptoms. Our results showed that when the patient with dementia was a parent, women alone were likely to have depressive symptoms, and when the patient with dementia was a spouse, both men and women were likely to have depressive symptoms. The generality of women having higher depressive symptoms than men was consistent with a previous series of studies using the CES-D to provide evidence that women caregivers score higher than men [18,19].

Depressive symptoms in women when the patient with dementia was a parent may be due to cultural factors. One of the cultural norms in Korea is filial piety, which entails that the eldest son and his wife repay their parents by helping them to live comfortably and helping them carry out any difficult tasks [20]. In a cross-cultural study of caregiving, it was reported that 55.0% of primary caregivers for a dementia-affected elder in Korea were daughters-in-law. In addition, the relationship between the parent-in-law and the daughter-in-law has a weaker emotional connection than others [21]. Korean families in modern society are still maintaining norms of Confucianism and a patriarchal system, despite changes such as nuclear familization and increased individualism. The cultural stipulation that the realm of family is the domain of women remains in place [22] Consequently, this role puts enormous stress on married women, and they struggle to balance child-rearing activities and professional careers with the additional task of providing care for their older parents [23]. For men, a job-oriented lifestyle has made it difficult for to participate in household chores or family issues [24].

Regardless of gender, when one’s spouse or significant other has dementia, there is a higher chance of depressive symptoms, and this could involve many factors. First, due to demographic changes in society, men have adopted caring roles more than ever before, for example, 44% of men in the USA and nearly 40% of men in Canada took the role of caregiving [25]. This phenomenon is no different in Korea as well. It is said that men are burdened not only by caring for patients with dementia, but also by the fact that housework is added to their burden [26]. Second, spouse caregivers may not have expected their role to be that of a caregiver. As explained above, the cultural norm of filial piety has made parents expect their children to provide care. However, when the role of caregiver is handed to the spouse, they may not be prepared for the role. Third, spouse caregivers are likely to have poorer personal health and lower social support than their adult children because of their age [27]. When the spouse has to take on a caregiver role, it results in spouses giving up most of their own lives to take care of their significant other [28]. It is also known that spouse caregivers are often emotionally fatigued and are required to prioritize care for their spouses rather than their own health [29]. The only treatment is early detection to prevent further worsening of dementia. This fact can make spouses feel anxiety and fear, and when dementia reaches a certain stage, spouses feel powerless [26].

In the study, when both men and women had white collar jobs, the likelihood of depressive symptoms was insignificant. This may be because better remuneration contributes to lower levels of burden. It could also be said that someone in a white collar job could develop more effective skills in managing the problems of care and personal stress. These factors may function as buffers to the stressor of caregiving [30]. In women, not having a job had the most significant effect in relation to employment status. It needs to be considered that the unemployed category also includes housewives. The role of a housewife in Korea is a mixture of a social role burden and a personal burden [31]. Because housekeeping and family labor are not socially recognized, when housewives can obtain a socially recognized job and integrate into society, they are more likely to feel satisfied [32]. This can be compared to men, in whom pink collar and blue collar jobs showed the most significant effects.

Both men and women who lived in rural areas had a higher chance of having depressive symptoms. This could be due to the lack of medical support in rural areas. Dementia caregivers living in rural settings experience increased challenges, such as far distances and limited options for health and supportive services, such as local dementia-friendly acute services [33]. In particular, the accessibility of medical and welfare facilities is a very important factor in the health of elderly people with dementia. However, in Korea, medical and welfare institutions are concentrated in large cities, and there is a shortage of qualified labor in rural areas. In addition, in smaller cities, it is difficult to secure manpower and space for centers focused on specific aspects of health; therefore, many health centers combine dementia projects with projects focused on other health issues, rendering it difficult to implement dementia-specific projects such as dementia screening and counseling [34].

The current study has several limitations. First, the severity of dementia and the duration of dementia are not known. Participants in this study may have been influenced by the severity of dementia. It is known that the more severe the dementia, the more the stress and the more likely family caregivers are to have severe depression. Second, although the PHQ-9, which has been tested for validity and reliability, was utilized in this study, the participants’ reports of depressive symptoms might still be subjective and imperfect. However, this survey was conducted by a trustworthy national institution, the KDCA, using skilled interviewers and a computer-based system. For this reason, PHQ-9 scores should be a good indicators of depressive symptoms. Third, it was not possible to adjust for other diagnoses of psychiatric disorders in caregivers. Fourth, as our study was cross-sectional in nature, a causal relationship could not be established. Despite these limitations, this study has several strengths. We used the most recent nationally representative database to determine associations. Therefore, the results are highly representative of the adult population in South Korea. In addition, we utilized the self-reported PHQ-9, an easy and valid tool for screening patients with depressive symptoms. To our knowledge, this is the first study using the PHQ-9 with KCHS data to determine the association between living with patients with dementia and family caregivers’ depressive symptoms.

## 5. Conclusions

The results of this study confirm an association between the presence of patients with dementia in a family and family caregivers’ depressive symptoms. In particular, when the patient with dementia is a spouse, both women and men are likely to have depressive symptoms, and when the patient with dementia is a parent, women are likely to have depressive symptoms. National-level policies are needed to support the family caregivers’ and provide necessary treatments.

## Figures and Tables

**Table 1:** General characteristics of study subjects.

	Depressive Symptoms (PHQ-9 ≥ 10)									
	Men (n = 186,183)					Women (n = 185,104)				
	Yes		No			Yes		No		
	N	%	N	%	*p* Value	N	%	N	%	*p* Value
Total (n = 371,287)	4474	(2.4)	181,709	(97.6)		7681	(4.1)	177,423	(95.9)	
**Relation with Dementia Patient**					<0.0001					<0.0001
Parent	192	(2.1)	8826	(97.9)		432	(4.7)	8730	(95.3)	
Spouse	82	(9.4)	793	(90.6)		73	(10.8)	606	(89.2)	
Other *	19	(3.3)	557	(96.7)		32	(5.1)	593	(94.9)	
None	4181	(2.4)	171,533	(97.6)		7144	(4.1)	167,494	(95.9)	
**Age**					<0.0001					<0.0001
19–39	972	(2.1)	44,289	(97.9)		2062	(4.2)	46,465	(95.8)	
40–49	594	(1.8)	31,526	(98.2)		876	(2.6)	33,268	(97.4)	
50–59	725	(1.9)	36,520	(98.1)		1118	(2.9)	37,348	(97.1)	
60–69	755	(2.1)	35,033	(97.9)		1244	(3.9)	30,531	(96.1)	
≥70	1428	(4.0)	34,341	(96.0)		2381	(7.4)	29,811	(92.6)	
**Region**					0.0466					0.3897
Metropolitan	1410	(2.5)	54,755	(97.5)		2495	(4.2)	56,802	(95.8)	
Rural	3064	(2.4)	126,954	(97.6)		5186	(4.1)	120,621	(95.9)	
**Educational level**					<0.0001					<0.0001
Middle School or Less	1804	(3.8)	45,690	(96.2)		3744	(5.9)	60,188	(94.1)	
High School	1361	(2.3)	57,612	(97.7)		1927	(3.7)	50,806	(96.3)	
University or Higher	1309	(1.6)	78,407	(98.4)		2010	(2.9)	66,429	(97.1)	
**Job Status**					<0.0001					<0.0001
White	515	(1.2)	41,412	(98.8)		976	(2.6)	36,299	(97.4)	
Pink	315	(1.6)	18,956	(98.4)		940	(3.1)	29,692	(96.9)	
Blue	1214	(1.5)	77,304	(98.5)		1133	(3.2)	34,705	(96.8)	
Unemployed	2430	(5.2)	44,037	(94.8)		4632	(5.7)	76,727	(94.3)	
**Household Income**					<0.0001					<0.0001
High	696	(1.3)	53,689	(98.7)		1405	(2.6)	53,651	(97.4)	
Middle high	777	(1.5)	49,642	(98.5)		1397	(2.9)	46,088	(97.1)	
Middle low	671	(2.0)	32,795	(98.0)		1219	(4.0)	29,128	(96.0)	
Low	2330	(4.9)	45,583	(95.1)		3360	(6.5)	48,556	(93.5)	
**Marital Status**					<0.0001					<0.0001
Married	2505	(1.9)	131,587	(98.1)		3638	(3.1)	115,382	(96.9)	
Not Married	1969	(3.8)	50,122	(96.2)		4043	(6.1)	62,041	(93.9)	
**Number of Family Member Living in the Same Household**					<0.0001					<0.0001
1	1067	(4.8)	20,954	(95.2)		2098	(7.1)	27,601	(92.9)	
2	1803	(2.4)	71,792	(97.6)		2517	(4.1)	59,396	(95.9)	
3	762	(1.9)	38,655	(98.1)		1392	(3.6)	36,862	(96.4)	
4	594	(1.6)	35,809	(98.4)		1141	(3.0)	37,196	(97.0)	
5+	248	(1.7)	14,499	(98.3)		533	(3.2)	16,368	(96.8)	
**Current Smoking**					<0.0001					<0.0001
Current Smoker	1870	(2.9)	63,350	(97.1)		799	(12.4)	5623	(87.6)	
Current Nonsmoker	2604	(2.2)	118,359	(97.8)		6882	(3.9)	171,800	(96.1)	
**Alcohol Use in the Past Year**					<0.0001					<0.0001
Yes	3063	(2.0)	152,712	(98.0)		5034	(3.5)	137,192	(96.5)	
No	1411	(4.6)	28,997	(95.4)		2647	(6.2)	40,231	(93.8)	
**Recipient of Basic Living Allowance**					<0.0001					<0.0001
Yes	674	(12.7)	4641	(87.3)		875	(15.6)	4722	(84.4)	
No	3800	(2.1)	177,068	(97.9)		6806	(3.8)	172,701	(96.2)	
**Year**					<0.0001					<0.0001
2018	2459	(2.6)	90,983	(97.4)		4137	(4.4)	90,283	(95.6)	
2019	2015	(2.2)	90,726	(97.8)		3544	(3.9)	87,140	(96.1)	

* Offspring and siblings.

**Table 2:** Associations between depressive symptoms (PHQ-9) and participants demographics.

Variables	Depressive Symptoms (PHQ-9 * ≥ 10)			
	Men		Women	
	OR	95% CI	OR	95% CI
**Relation with Dementia Patient**				
Parent	1.07	(0.92–1.24)	1.47	(1.32–1.62)
Spouse	2.65	(2.06–3.32)	2.28	(1.77–2.93)
Other *	1.18	(0.74–1.89)	1.00	(0.69–1.43)
None	1.00		1.00	
**Age**				
19–39	1.00		1.00	
40–49	1.02	(0.91–1.14)	0.58	(0.53–0.63)
50–59	0.81	(0.72–0.91)	0.51	(0.46–0.55)
60–69	0.60	(0.53–0.69)	0.47	(0.42–0.52)
≥70	0.72	(0.63–0.82)	0.60	(0.54–0.67)
**Region**				
Metropolitan	1.00		1.00	
Rural	0.91	(0.85–0.97)	0.91	(0.86–0.95)
**Educational level**				
Middle School or Less	1.76	(1.58–1.95)	1.91	(1.73–2.11)
High School	1.34	(1.23–1.47)	1.50	(1.39–1.62)
University or Higher	1.00		1.00	
**Job Status**				
White	1.00		1.00	
Pink	1.08	(0.93–1.24)	0.95	(0.87–1.05)
Blue	0.89	(0.79–1.00)	0.83	(0.75–0.92)
Unemployed	2.33	(2.08–2.62)	1.41	(1.30–1.53)
**Household income**				
High	1.00		1.00	
Middle high	1.10	(0.99–1.23)	1.01	(0.94–1.09)
Middle low	1.23	(1.09–1.38)	1.26	(1.16–1.37)
Low	1.83	(1.64–2.05)	1.68	(1.54–1.83)
**Marital Status**				
Married	0.73	(0.67–0.80)	0.71	(0.67–0.75)
Not Married	1.00		1.00	
**Number of Family Member** **Living in the Same Household**				
1	1.00		1.00	
2	0.73	(0.66–0.80)	1.02	(0.95–1.10)
3	0.78	(0.69–0.87)	1.07	(0.98–1.17)
4	0.78	(0.69–0.89)	0.99	(0.90–1.09)
5+	0.76	(0.64–0.89)	0.95	(0.85–1.07)
**Current Smoking**				
Current Smoker	1.53	(1.44–1.64)	2.89	(2.66–3.14)
None Current Smoker	1.00		1.00	
**Alcohol Use in the Past Year**				
Yes	0.57	(0.53–0.61)	0.71	(0.68–0.75)
No	1.00		1.00	
**Recipient of Basic Living Allowance**				
Yes	2.34	(2.11–2.58)	2.23	(2.05–2.43)
No	1.00		1.00	
**Year**				
2018	1.00		1.00	
2019	0.81	(0.76–0.86)	0.91	(0.87–0.95)

* Offspring and siblings.

**Table 3:** Subgroup analysis of the association between relation with dementia patient and caregiver’ depressive symptoms stratified by sociodemographic variables.

Variables	PHQ-9 ≥ 10						
	Relation with Dementia Patient						
	None	Parent		Spouse		Others *	
	OR	OR	95% CI	OR	95% CI	OR	95% CI
**Men**							
**Age**							
19–39	1.00	0.97	(0.70–1.35)	3.29	(1.18–9.18)	1.51	(0.36–6.26)
40–49	1.00	1.17	(0.82–1.68)	1.08	(0.14–8.04)	0.86	(0.11–6.78)
50–59	1.00	0.98	(0.72–1.33)	3.29	(0.99–10.92)	0.57	(0.07–4.44)
60–69	1.00	1.25	(0.91–1.72)	7.74	(4.17–14.37)	1.74	(0.70–4.33)
≥70	1.00	1.12	(0.73–1.70)	2.34	(1.77–3.09)	1.13	(0.59–2.15)
**Job Status**							
White	1.00	0.85	(0.53–1.35)	2.89	(0.69–12.12)	1.55	(0.21–11.37)
Pink	1.00	1.14	(0.69–1.88)	5.25	(1.21–22.75)	1.33	(0.18–9.91)
Blue	1.00	1.30	(1.01–1.68)	4.74	(2.97–7.58)	1.67	(0.73–3.80)
Unemployed	1.00	1.02	(0.81–1.27)	2.22	(1.67–2.95)	0.97	(0.52–1.81)
**Region**							
Metropolitan	1.00	0.81	(0.60–1.09)	2.38	(1.45–3.90)	0.60	(0.19–1.93)
Rural	1.00	1.19	(1.00–1.42)	2.68	(2.04–3.52)	1.44	(0.86–2.42)
**Educational level**							
Middle school or less	1.00	1.08	(0.81–1.44)	2.52	(1.88–3.38)	1.19	(0.62–2.28)
High school	1.00	1.05	(0.81–1.35)	2.54	(1.46–4.40)	1.06	(0.42–2.65)
College or over	1.00	1.13	(0.86–1.42)	3.86	(2.06–7.21)	1.39	(0.50–3.82)
**Income**							
High	1.00	0.75	(0.52–1.08)	4.28	(1.94–9.45)	1.54	(0.37–6.37)
Middle high	1.00	0.97	(0.69–1.35)	2.74	(1.08–6.90)	1.24	(0.30–5.07)
Middle low	1.00	1.49	(1.07–2.08)	1.94	(0.78–4.82)	1.60	(0.58–4.39)
Low	1.00	1.18	(0.93–1.49)	2.65	(2.02–3.48)	1.03	(0.55–1.92)
**Women**							
**Age**							
19–39	1.00	1.65	(1.37–1.99)	2.63	(1.01–6.84)	0.69	(0.16–2.88)
40–49	1.00	1.20	(0.93–1.56)	0.65	(0.08–4.87)	0.99	(0.13–7.33)
50–59	1.00	1.59	(1.31–1.94)	1.74	(0.61–4.95)	1.40	(0.49–3.96)
60–69	1.00	1.53	(1.19–1.97)	2.13	(1.23–3.70)	1.50	(0.82–2.72)
≥70	1.00	1.17	(0.84–1.63)	2.38	(1.73–3.27)	0.78	(0.44–1.38)
**Job Status**							
White	1.00	1.37	(1.05–1.79)	1.65	(0.39–6.90)	2.13	(0.65–6.92)
Pink	1.00	1.49	(1.16–1.91)	2.54	(0.99–6.50)	1.94	(0.77–4.86)
Blue	1.00	1.63	(1.25–2.12)	2.11	(1.16–3.85)	0.54	(0.17–1.74)
Unemployed	1.00	1.44	(1.25–1.66)	2.32	(1.72–3.13)	0.92	(0.59–1.45)
**Region**							
Metropolitan	1.00	1.27	(1.05–1.52)	3.21	(2.02–5.09)	0.76	(0.35–1.64)
Rural	1.00	1.57	(1.39–1.78)	2.01	(1.48–2.71)	1.09	(0.72–1.65)
**Educational level**							
Middle school or less	1.00	1.34	(1.11–1.62)	2.18	(1.64–2.91)	1.13	(0.75–1.71)
High school	1.00	1.60	(1.35–1.90)	1.92	(0.92–4.02)	0.51	(0.16–1.62)
College or over	1.00	1.44	(1.21–1.71)	3.64	(1.74–7.61)	0.91	(0.28–2.96)
**Income**							
High	1.00	1.31	(1.07–1.61)	3.02	(1.30–7.00)	0.47	(0.06–3.39)
Middle high	1.00	1.68	(1.37–2.06)	1.62	(0.50–5.24)	0.95	(0.30–3.05)
Middle low	1.00	1.45	(1.14–1.84)	2.18	(1.04–4.54)	0.92	(0.33–2.53)
Low	1.00	1.45	(1.20–1.75)	2.32	(1.73–3.11)	1.07	(0.70–1.64)

* Offspring and siblings.

## Data Availability

Publicly available datasets were analyzed in this study. This data can be found here: [https://chs.cdc.go.kr/chs/index.do].
